# Impact of Plantation Induced Forest Degradation on the Outbreak of Emerging Infectious Diseases—Wayanad District, Kerala, India

**DOI:** 10.3390/ijerph19127036

**Published:** 2022-06-08

**Authors:** Kakoli Saha, Debjani Ghatak, Nair Shruti S. Muralee

**Affiliations:** 1 Department of Planning, School of Planning and Architecture, Bhopal 462030, India; 2 Department of Geography, Texas A&M University, College Station, TX 77843, USA; debjani.ghatak@tamu.edu; 3 Madhya Pradesh Rurban Mission, Government of India, Bhopal 462030, India; shruti.nair1706@gmail.com

**Keywords:** LULCC, emerging infectious disease, India, Kerala, KFD, dengue, GIS, remote sensing, leptospirosis, Wayanad, forest degradation, plantation

## Abstract

**Simple Summary:**

The emerging infectious diseases (EIDs) have increased recently due to forest degradation. This research is an attempt to understand the direct and indirect impact of forest degradation on infectious disease outbreaks in a specific context of the Wayanad district in Kerala, India. The study was done in two parts. In the first part, land use and land cover change of the Wayanad district was analyzed for the period of 1950 to 2018. The result of the analysis shows that a significant amount of forest has been converted into agricultural and forest plantations over the time. The second part involves understanding the impact of plantations on the outbreak of EIDs. We have employed GIS tools, remote sensing data, extensive field work and disease data to discover the relationship between the LULCC and disease outbreak. It was found that cases of EIDs were high in those gram panchayats where forests were encroached by plantations.

**Abstract:**

The world has been facing a pandemic owing to COVID-19. We have also seen the geographic expansion and outbreaks of other emerging infectious diseases (EID) in recent years. This paper investigates the direct and indirect effects of land use land cover change (LULCC) on EID outbreaks in the context of Wayanad District of Kerala, India. Wayanad is in the vulnerable tropical forested region, and it is named as one of the four environmental change hotspots. The focus of this project is mainly three EIDs prevalent in this region: Kyasanur forest disease (KFD), Dengue and Leptospirosis. Our results, based on topographical map, remote sensing and extensive field work, show that the natural forest in Wayanad was replaced with agriculture and forest plantation during 1950–2018. This paper further suggests that encroachment of forest by forest plantation causes the human–animal conflict resulting in the outbreak of KFD cases. Our analysis reveals that a high number of Dengue cases is found in the forested regions of the district and over the adjacent human-made agriculture plantation areas. High and medium number of Leptospirosis cases contain a high portion of land area devoted to paddy cultivation and agricultural plantation. In summary, the results clearly show the linkage between the outbreak of above mentioned EIDs and LULCC in the context of Wayanad district, Kerala. We also discuss in detail the causal pathway involving human–environmental dynamics through which plantation leads to the outbreak of KFD. Replacing forests with plantations poses an alarming threat of disease outbreak in the community.

## 1. Introduction

The fatal impact of pandemics is not a matter of scientific discussion anymore and is not just constrained to the scientific literature. The world has been facing a pandemic due to the outbreak of COVID-19 during the preparation of this manuscript. Emerging infectious diseases (EIDs) refer to the suite of infectious diseases whose occurrences have affected civilization more frequently recently (in the past 20 years) and could increase soon (as defined by World Health Organization). We have seen more frequent outbreaks of EIDs recently. Many of the EIDs are Zoonotic types of diseases (transmitted between animal and human) [1,2]. Scientists have warned us about the impact of land-use and land-cover change (LULCC), which leads to the spread of diseases. More specifically, existing literature has suggested that LULCCs have led to the outbreak of EIDs [3,4,5,6]. 

LULCCs occur because of human actions, as well as due to natural causes. LULCC has long-term effects as well; for example, forest degradation results in the loss of biodiversity and poses a threat on the supply of food, water, and shelter to the ecosystem [7,8,9,10]. Tropical forests are well-known for their significant role in containing one half to two-thirds of the world’s plant and animal species [11] and hence, they are rich in biodiversity. Tropical forests have been facing changes due to deforestation for the purpose of logging, plantations, agricultural expansion, and expansion of human settlements [9].

It is found that the forest fragmentation enhances the concentration of howler monkeys who are the hosts of South American yellow fever [12]. The current pandemic is also suggested to be associated with the deforestation in Southeast Asia [12]. Encroachment of Nipah virus from bats to pigs and humans occurred in Malaysia due to deforestation [13]. Ebola virus disease causing severe and fatal hemorrhagic fever in humans has been linked with forest fragmentation and deforestation [14,15]. However, there are many things that are still unknown to us; e.g., the impact of deforestation on the spread of pathogens in birds [16]. Birds can be efficient in spreading infectious agents to humans and other wildlife [16]. 

Zoonotic infections, which had historically affected only animals, are now transmitted to people due to increased contact between human and animals [17]. Wilcox et al. (2006) [18] emphasized the relationship between deforestation and EIDs. For example, some logging processes within forest areas may increase in malaria mosquito breeding sites through standing water. The loss of forest cover also introduces human-induced species, which make these areas vulnerable to the emergence and spread of disease through new transmission pathways [19,20]. Gottwalt (2013) [21] emphasized the fact that the deforestation directly impacts human health through the increase in infectious diseases.

The goal of this paper is to explore the direct and indirect impact of LULCC on EID outbreaks in the context of the Wayanad district of Kerala (one of the states in India along the Western Ghats Mountain). Wayanad (11.45° N–11.97° N, 75.5° E–76.5° E) is chosen for this purpose because of its geographic location in a vulnerable tropical forested region and because it is named as one of the four environmental change hotspots of Kerala [22,23]. The Western Ghats extend over an area of 164,280 km^2^ between 8–22.43° N and 72.91–78.18° E [24]. The Western Ghats lost approximately 35% of total forest cover between 1920 and 2013 [25]. Loss of forest cover is well documented not only over just one area, but also at many parts of Western Ghats [26,27,28,29,30]. Kerala (one of the Indian states along the Western Ghats Mountain) has lost 5000 ha of forest annually from 1940–1970 [26]. The annual deforestation rate is 1.4% for the period from 1972 to 1982 in Kerala [31]. Loss of forest in Kerala is evident through the satellite image-based analyses [32]. According to the Climate Vulnerability Assessment report (2019–2020) [33] published by the government of India, Wayanad district of Kerala is moderately vulnerable with a Vulnerability Index between 0.559–0.629. Vulnerability Index is a measure based on a set of common indicators to assess and compare the relative vulnerabilities of Indian states/districts/blocks [33]. There were 18 indicators considered for the assessment, which include relatively low forest cover per 1000 population and a number of vector and waterborne diseases per 1000 population. Considering the vulnerability and possible linkage between LULCC and diseases, the Wayanad district is selected as the case study districts. Thus far, scholars have studied EIDs of Kerala in relation to domestic and environmental factors [34]; women health in rural Kerala [35]; economic impact [36]; and response to illness [37]. This paper examines the contribution of LULCC to the emergence of EIDs in the context of Wayanad district, Kerala.

## 2. Background

### 2.1. Study Area

**Figure 1:**
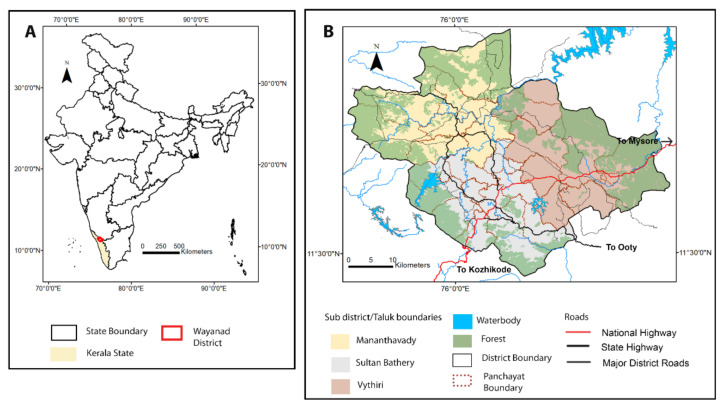
(**A**) Location of Wayanad district in Kerala within the Indian boundary. (**B**) Base map of Wayanad district showing major subdistricts and land use/covers.

Figure 1A shows the geographical location of the state of Kerala and Wayanad District in the map of India. Wayanad district covers an area of 2120 sq. km and 40% of its area is covered by forests. Forest cover has become absent from the central part (Figure 1B). The district is subdivided into three sub districts namely, Mananthawady, Sultan Bathery and Vythiri (Figure 1B). The subdistricts are further divided into 26 g panchayats (rural administrative boundary) (Figure 1B). Wayanad is the least populated district of Kerala, with a total population of 817,420 [38], which is 2.45% of the total population of the state. It is the less urbanized district, with a very high rural population and with the highest percentage of state forest dwellers (26%). 

### 2.2. Historical Perspective of LULCC in Kerala with a Focus on Wayanad

Anoop et al. (2020) [23] reported large scale land-cover transformation in Wayanad after the arrival of British during 1800. At first, they used fire as a tool to clear the forest. Subsequently, plantations of coffee, cinchona, tea, and hardwood trees were established by removing timber from the forests. Timber was also used as resources for domestic purposes and for the construction of roads, bridges, and settlements. Road networks were expanded to facilitate the transportation of natural resources [23]. After converting forests to plantation areas, they brought hundreds of workers to Wayanad from neighboring regions. This added additional pressure on the forests of Wayanad [23]. 

### 2.3. Outbreak of EIDs in Kerala with a Focus on Wayanad

Kerala has been experiencing public health challenges due to the outbreak of EIDs [37]. Three EIDs are selected for this study, and they are KFD, Dengue and Leptospirosis. Dengue disease causes fever, and the different virus serotypes (DENV-1, DENV-2, DENV-3, DENV-4) are transmitted through the infected *Aedes aegypti* and *Aedes albopictus* female mosquitoes [39]. The strain of the virus, vulnerability of human population and density, behavior and competence of the vector population may produce a Dengue epidemic [40]. During the last five decades, Dengue cases have increased 30-fold [41] globally. Dengue patients may experience more fatal dengue hemorrhagic fever (DHF) and Dengue Shock Syndrome (DSS).

Leptospirosis disease caused by bacteria *Leptospira* causes fever and may lead to complications on multiple organs. Leptospirosis is primarily prevalent in the southern, central, eastern and western parts of India, but few cases are reported over North India recently [42]. Though Leptospirosis disease is underdiagnosed and underestimated, tens of millions of people are infected annually with 5–25% fatality rates [43]. Leptospirosis, which has appeared as a major public health issue in Kerala, was constrained only in some areas of Alappuzha and Kottayam districts in the 1990 [44]. A very high number of deaths are reported consistently for the last few years in the state due to Leptospirosis [45].

Kyasanur forest disease (KFD) is another infectious disease that is prevalent in Kerala, specifically in the Wayanad district. Initially, KFD cases were more common in other states of India (e.g., Karnataka) [46,47], but the outbreak of this disease is reported in Kerala as well. KFD, known as “Monkey fever,” is a zoonotic viral hemorrhagic fever. This is a tick-borne disease caused by the Kyasanur forest disease virus belonging to Flaviviridae family [48]. Five hundred cases are reported annually with 10% mortality in the Western Ghats region of South Indian [49]. A study based on household survey suggests 69% of respondents were affected by KFD [50].

## 3. Data and Methods

**Figure 2:**
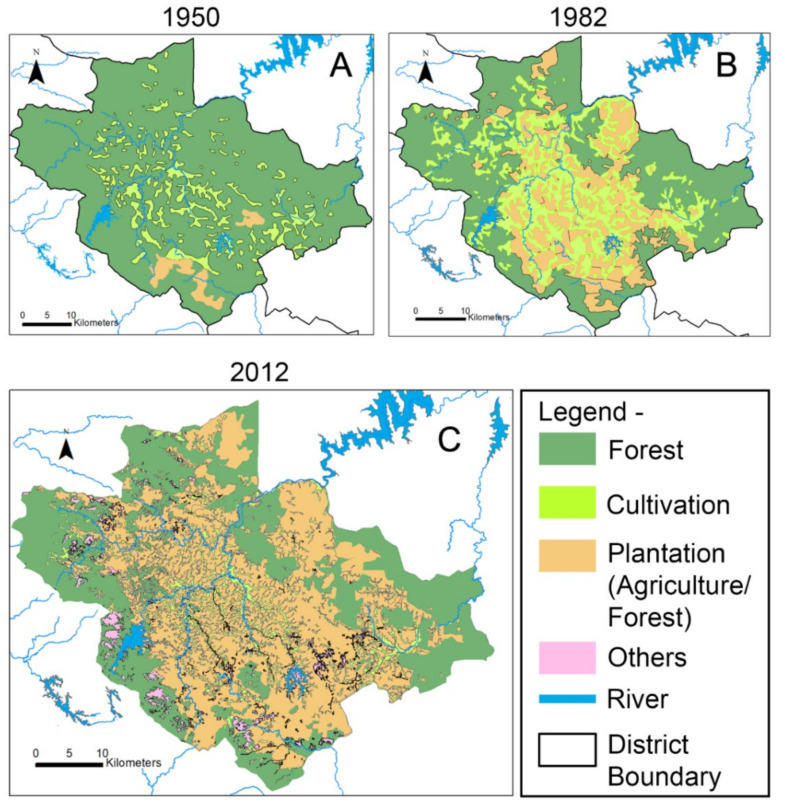
Land use land cover change of Wayanad district from 1950 to 2012; (**A**) land use land cover till 1950; (**B**) land use land cover till 1982; and (**C**) land use land cover till 2012.

The change in land use land cover has been analyzed from 1950 to 2018. The map of 1950 was the earliest LULC map that we had access to. To observe significant change in the LULC, considerable time interval was chosen (1950, 1982, 2012) (see Figure 2). After 2012, the latest LULC map that was available belongs to 2018. The following section elaborates the method of acquiring the maps.

To address the lack of reliable data in 1950, a topographical map has been digitized onscreen in GIS platform to produce the land use land cover map of 1950 (Figure 2A). Unrestricted topographical maps published by Survey of India are available for sale (Survey of India, Topographical map of Wayanad district, Kerala, surveyofindia.gov.in, accessed on 4 March 2022). The standard topographical maps are available at the scale of 1:25,000, 1:50,000, and 1:250,000. In this study, a topographical map at the scale of 1:250,000 has been used. A land use land cover map of 1982 (Figure 2B) at the scale of 1:250,000 has been produced by digitizing the map published in the report by the French Institute of Pondicherry [51]. 

‘Bhuvan’ is a geoportal of Indian Space Research Organization (ISRO), which makes recent Indian Earth Observation data products available for the users. ‘Bhuvan’ also provides interpreted satellite imageries, thematic data, and other services. The thematic maps in the geoportal include land use and land cover data for the entire India at the scale of 1:250,000. Therefore, the land use and land cover map of 2012 (Figure 2C) has been acquired from ‘Bhuvan’ (www.bhuvan.nrsc.gov.in, accessed on 4 March 2022). Subsequently, they are georeferenced and have been digitized in GIS platform to create vector layers. While digitization, projected coordinate system like Universal Transverse Mercator (UTM) zone 43 N has been used for horizontal referencing and World Geodetic System—WGS 84 has been used for vertical referencing.

**Figure 3:**
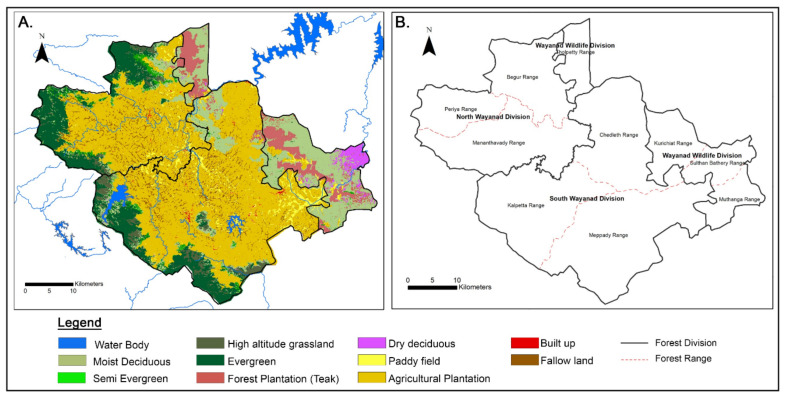
(**A**) Detailed LULC of Wayanad district of 2018 showing forest and agricultural plantations. (**B**) Forest divisions and ranges in Wayanad district.

The Landsat 8 image for the year 2018 was downloaded from the online portal of USGS. The image was of 30 m spatial resolution (Figure 3A). It was used to prepare land use land cover maps using the image analysis algorithms available in Google Earth Engine. The supervised classification technique was used to classify the image into different land use types. To geo-reference the image, a horizontal referencing system such as Universal Transverse Mercator (UTM) zone 43 N was used. To assign the vertical datum, World Geodetic System (WGS) 84 was used. Supervised classification requires identification of ‘training sets’, which are set of pixels that characterize LULC categories based on reflectance pattern. In this research, training sites for signature generation were developed using ground truth data. Multiple training sites were selected for each LULC category to ensure that all pixels are assigned to their respective classes. The image analysis software performed statistical analysis of reflectance value of pixels in all bands and assigned them to pre-defined classes based on their statistical characteristics [52]. 

The image was then classified using Support Vector Machine (SVM) classifier available in Earth Engine. SVM draws a decision boundary which is a hyperplane between any two classes to classify them. Finally, the eleven LULC classes, namely evergreen, high altitudinal grassland, moist deciduous, dry deciduous, teak plantation, waterbody, mixed plantation (coffee, rubber, areca nut palms, cocoa, pineapple), paddy cultivation, tea plantation, build up area and fallow land were identified. Since this study focused on impact of plantations, mixed plantation and tea plantation were merged to create a new class namely agricultural plantation (Figure 3A). Teak plantation was renamed as forest plantation (Figure 3A). The accuracy assessment is an integral part of any image analysis process. In this research, accuracy assessment was not performed separately as the training sites were selected from ground truth data. 

Primary data collection was integral part of this research to examine the causal pathway of disease outbreak through human–animal interaction. An informal interaction was held with Chief Conservator of Forests, Mr. Dr. P Pugazhendi (Indian Forest Service) in February 2020. The set of questions as asked are attached in Supplementary Materials. The updated data on the number of cases of EIDs over Wayanad district was collected from District Medical Office (Health), Wayanad, Kerala. 

## 4. Results

### 4.1. LULCC in Wayanad District of Kerala

Overall, 1950 is the earliest year when LULC data on Wayanad is available. Figure 2 and Table 1 present LULCC in Wayanad. Please check Section 3 for the details about the source of these LULC maps. Despite land cover transformation under the British (see Section 2.1) during the colonial rule, 85% of the total area of Wayanad was under forest cover till the 1950s (Figure 2A). Forest cover is reduced from 1811.35 sq km in 1950 to 1064.68 sq km in 1982 (Figure 2B). The reason behind the ~41% loss of forest cover between 1950–1982 lies in the Government policy. In the late 1950s, the State Government of Kerala made a contract with an industry (Aditya Birla Group) to start a pulp factory for ‘Grasim Gwalior Rayons’ at Mavoor, an agrarian village, located in the adjacent district of Wayanad [53]. One of the raw materials for the production, bamboo was supplied from the Wayanad district at nominal price resulting in the destruction of lush bamboo forest in the district. Large tracks of natural forests were laid to waste. Additionally, cultivation and plantation increased 107% and 729%, respectively, between 1950 and 1982 (Table 1) as the tropical forests were converted into industrial monoculture plantations (Figure 2A,B).

The main cause of the loss of forest cover between 1982 and 2012 is forest fires. There were 316 fire incidents, mainly due to human-induced causes, in the Wayanad Wildlife Sanctuary between 2001 and 2011 [54]. As a result, the forest cover has been reduced by 2012 (Figure 2C). The decrease in cultivation from 1982 to 2012 happened as the multi-cropping pattern is replaced by mono-cropping/plantation-type cultivation (Figure 2B,C). In summary, there has been a ~51% decrease in forest cover occurred during 1950–2012. Whereas a ~1329% increase in plantation cover occurred during the same time (Table 1). 

Further analysis involved the preparation of a detailed LULC map of 2018 with the help of field survey (Figure 3). A field survey was required, as satellite-based imagery often fails to differentiate between natural forest and plantation forest. Spatial extension of plantation area has been increased between 2012 and 2018, and consequently, forest area has decreased (Figure 3A). Figure 3A also shows the distribution of different mono-culture plantations (please see the details of the methodology in Section 3). Field survey confirmed the following spatial pattern. Tea, coffee, rubber, areca nut palms, cocoa, and pineapple are cultivated under agriculture plantations [23]. Forest plantation specifically includes teak, teak and softwood, eucalyptus, rosewood, bamboo, sandalwood, and other hardwood. Under forest plantation, patches of teak plantation have been mapped from satellite imagery. This plantation is concentrated at the north and eastern sides of the district, mainly over the Wayanad Wildlife Division (compare Figure 3A,B). Teak plantation covers 49.61% of total plantation areas (Figure 3A). On the other hand, tea plantation is scattered over the north, west, and southern sections of the district. Most of the district is covered by agricultural plantations as mentioned above. 

The LULC map is discussed in relation to forest divisions and forest range to understand the extent and degree of change in land use and land cover (LULCC) within forest areas of Wayanad. To understand the extent of plantation, a forest division wise summary table was constructed (Table 2). Both North and South Wayanad forest divisions have more land area under plantation than natural forest. Table 2 clearly shows that the Wildlife Division also has high number of land area (33.04%) under plantations. On average, 51.44% area of entire district is under human-made plantation, while only 38.36% area is under natural forest in 2018 (Table 2). 

### 4.2. Correlation between LULCC and Disease Outbreak in Wayanad

To investigate the direct or indirect impact of LULCC on the spread of EIDs, a list of confirmed cases of three selected EIDs from Wayanad district is shown in Table 3 (data acquired from the directorate of health services). Three EIDs are KFD, Dengue and Leptospirosis. Since the earliest available data on EIDs for Wayanad district goes back to 2013, the following section examines the association between LULCC and spread of EIDs for the years after 2013.

#### 4.2.1. LULCC and KFD in Wayanad District

**Figure 4:**
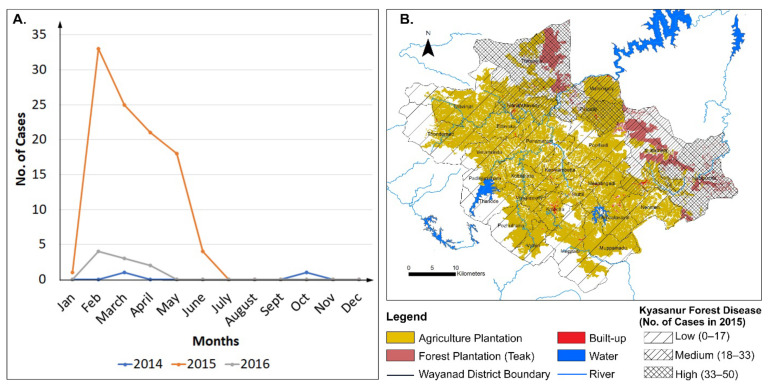
(**A**) Number of KFD cases in Wayanad for the year 2014, 2015 and 2016. (**B**) KFD cases in 2015 at Gram Panchayat level (rural administrative units) superimposed on a plantation map showing Correlation between LULCC and KFD cases.

Figure 4A shows the number of KFD cases in Wayanad for each month of the year 2014, 2015 and 2016. KFD generally occurs during winter and spring months with some exceptions in the fall season.

**Figure 5:**
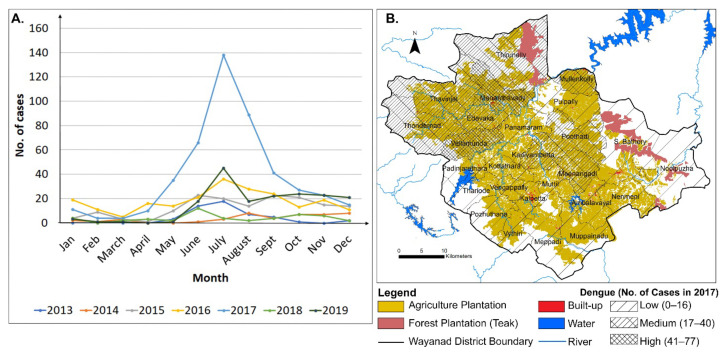
(**A**) Month wise dengue cases in Wayanad district for the time 2013–2019, and (**B**) thematic map showing Gram Panchayat wise dengue cases in 2017 superimposed on plantation map.

A thematic map was prepared by superimposing KFD cases in 2015 at Gram Panchayat level (rural administrative units) on a plantation map (Figure 5B). The maximum number of cases was reported in Wayanad during 2015; hence, the year 2015 was selected for this analysis (Table 3). Pupally, Tirunelly and Noolpuzha Gram Panchayats fall under the category of highest number of cases (34–50). On the other hand, Sultan Bathery, Mullenkolly and Manathavady Gram Panchayats fall under the category of medium number of cases (18–33). The rest of the Gram Panchayats belong to the category of lowest number of cases. This analysis suggests that Teak Plantation is predominant and widespread in the Gram Panchayats, which exhibit medium to high numbers of KFD cases (Figure 4B).

In other words, Tirunelly, Noolpuzha and Sultan Bathery Gram Panchayats (rural administrative units) overlap with “Wayanad Wildlife Divisions” (Figure 3B and Table 2) which is heavily covered by teak plantation. Supplementary Figure S1 shows the similar relationship that the high number of KFD cases occurs in 2020 over the same areas of the Wayanad district where teak plantation is prevalent. Overall, 2020 is the year when the number of KFD cases were reported as the second highest (Table 3).

#### 4.2.2. LULCC and Dengue in Wayanad

Figure 5A shows the monthly number of dengue cases in Wayanad for the time 2013–2019. Dengue generally occurs during summer and monsoon months. To understand the correlation between LULCC and dengue fever outbreak spatially, a thematic map showing Gram Panchayat wise dengue cases in 2017 is presented (Figure 5B). The year 2017 was chosen as the highest number of dengue cases were reported in that year (Table 3). The thematic map is superimposed on the spatial map of agriculture and forest plantation (Figure 5B). The highest number of dengue cases (41–77) was reported from Mananthavady, Vellamunda gram panchayats. The medium number of dengue cases (17–40) were recorded from the Thavinjal, Edavaka, Thirunelly, Thondernad, Mullenkolly, Kaniyambetta, and Poothadi gram panchayats (Figure 5B). The rest of the gram panchayats belong to the low number of dengue cases category. A major portion of land areas in the gram panchayats with high and medium number of dengue cases are covered with both natural forest and human-made plantations. In summary, this analysis suggests that the dengue outbreak happened over the forested regions of the district and over the adjacent human-made agriculture plantation areas. Our results verify that this relationship holds true for the year 2016 (Supplementary Figure S2) when the number of dengue cases was reported as the second highest (Table 3 and Figure 5A). 

#### 4.2.3. LULCC and Leptospirosis in Wayanad District

**Figure 6:**
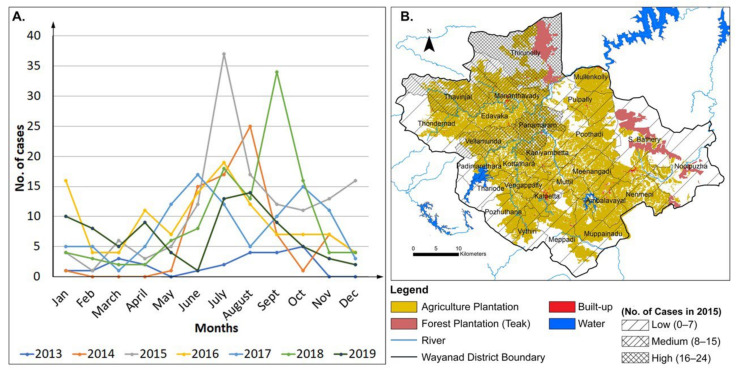
(**A**) Month-wise Leptospirosis cases in Wayanad district. (**B**) Correlation between LULCC of Wayanad and Leptospirosis disease.

Figure 6A shows the monthly number of Leptospirosis cases in Wayanad for the time 2013–2019. Leptospirosis generally occurs during monsoon and post monsoon months. To understand the spatial relationship between LULCC and Leptospirosis disease, thematic map showing Leptospirosis cases were prepared using Gram Panchayat-wise Leptospirosis cases in 2015 (Figure 6B). The year 2015 is chosen because the highest number of Leptospirosis cases were reported in that year (Table 3). The thematic map was overlaid on the top of the plantation map showing agricultural and forest plantations (Figure 6B). The highest number of Leptospirosis cases (16–24) were reported from the Thirunelly and Panamaram gram panchayats. The medium number of Leptospirosis cases (8–15) were reported from the Mananthavady, Thavinjal, and Vellamunda gram panchayats (Figure 6B). Gram panchayats with high and medium numbers of Leptospirosis cases contain high portion of land area devoted to paddy cultivation and agricultural plantation (Figure 6B). The rest of the gram panchayats belong to the low number of Leptospirosis cases category. Our results confirm that this relationship holds true for the year 2020 (Supplementary Figure S3) when the number of Leptospirosis cases was reported as the second highest (Table 3 and Figure 6A).

## 5. Discussion

Monkeys play the role of host with KFD virus-infected ticks *Haemaphysalis spinigera*, especially *Macaca radiata* (the Bonnet Monkey) and *Semnopithecus entellus* (Black faced langurs) [48]. Humans get infected from these infected ticks through monkey–human contact [48]. This type of contact happens during human–monkey conflict. The number of such conflict cases are recorded by the forest department of Kerala. A high number of cases were recorded in 2015, which was also the year with maximum KFD outbreaks in Wayanad district (Supplementary Table S1). 

Section 4.1 specifically shows how the deciduous forest in Wayanad Wildlife division is encroached by teak plantation (Figure 3A). The monoculture of teak plantation destroyed the local biodiversity. In Wayanad Wildlife Divisions, the plantation has only 70 plant species, whereas the species recorded in natural forest represented 84 genera in 46 families [48]. Thus, LULCC provided an opportunity for the extensive spread and establishment of various invasive plants. *Lantana(L.) camara* and *Senna spectabilis* are growing extensively in the wildlife division [55]. The invasive plants affect the composition and structure of native vegetation and result in its regeneration [23,55]. *L. camara* creates a barrier between the monkeys and their food sources [55]. This fact was confirmed by the Chief Conservator of Forests, Mr. Dr. P Pugazhendi (Indian Forest Service). He confirmed the fact that scarcity of resources like water and food inside the forest drives the animals away from the forest. Thus, this causes human–animal conflict and Wayanad is no exception. Furthermore, Chandran et al. (2016) [48] reported eight dead monkeys. 

Another reason for KFD outbreak is the encroachment of forest area. Encroachment inside the wildlife divisions is the constant concern of authorities (Kerala Forest Statistics, 2018). Tribes are most vulnerable to KFD infected ticks because of their forest dependent livelihood [46]. High abundance of *Haemaphysalis spinigera* tick species has been reported from the forest floor of Wayanad district in 2015 [46,48]. Most of the victims of this disease are from tribal areas, and all the reported deaths occurred among tribal people in 2015 [48]. However, the accurate number of cases is not reported for KFD at many times, and as a result, the number of cases remains underreported for KFD (Figure 4A).

Our results from Section 4.2.2 on the association between LULCC and dengue outbreak are complementary with the existing literature showing the impact of deforestation on the outbreak of vector-borne dengue disease over Mizoram (North-east state of India) [56]. Vector replication is favored in deforested regions by interrupting the ecological balance [3]. Vittor et al. (2006, 2009) [12,57] showed how deforestation affects the spread of the vector-borne disease in the Amazon by altering the vector breeding sites and by the changes in the microclimate suitability for parasite development. The risk of exposure to Dengue depends on the vector-to-host ratio, which varies with the land-use and landcover in Hawai [58]. Sarfaz et al. (2012) [59] suggested that the spatio-temporal correlation between vector larval density and land-use type should be considered for Dengue habitat mapping.

## 6. Conclusions

This paper examined the LULCC over the Wayanad district of Kerala during 1950–2018 and its impact on the outbreaks of EIDs. A combination of topographical maps, satellite-based data analyses and extensive field work shows that natural forest in Wayanad is replaced with agriculture and forest plantation. From 1950 to 2018, there has been 62% reduction in forest cover whereas 1800.75% increase in area under plantation (Table 1). 

Results based on the examination of Wayanad (Kerala) are complementary to the existing literature in the context of other parts of India and Kerala [29,60], which suggests the replacement of natural forests with agriculture and forest plantations. Capturing LULCC is difficult in India as “Forest cover” defined by the Forest Survey of India fails to distinguish between “Natural” forest versus human-made plantation [61]. Ravindranath et al. (2014) [62] pointed out how the forest cover is over-reported in India by including plantation and fruit orchards along with the under-reporting of deforestation [61]. Scientists strongly made the objection of considering plantation as “forest” by stating “plantations are not forests” [63] and “biological deserts” [64]. Plantations in most cases include single species stands; thus, it reduces the diversity. Monoculture in plantations is more vulnerable as this enhances the chances of pest/pathogen infestation/load [65]. Therefore, this paper examines how the expansion of plantation may have caused an outbreak of EIDs in the district of Wayanad.

This study clearly demonstrates that replacement of forest with both agriculture and forest plantation is associated with the outbreak of three EIDs (KFD, Dengue and Leptospirosis) as examined here. Furthermore, our results suggest specific pathways that the replacement of deciduous forest with the teak plantations over the northern and eastern parts of Wayanad results into the human–animal (monkey) conflict. Consequently, monkey population playing the host of KFD infected tick spreads the KFD virus in neighboring human settlements. Encroachment of forest areas due to the intrusion of human settlements inside the forest and over the adjacent areas of forests also makes the human population vulnerable to KFD disease. 

The obtained results also suggest the spread of dengue disease over the northern and southern parts of the district, which has natural forest cover as well as agriculture plantation areas. In Wayanad (Kerala), plantation areas surrounding the forest make the plantation laborers vulnerable to Dengue disease by increasing the human contact and mosquito biting rates. Leptospirosis also spreads through the human-animal contact as wild mammals get infected with Leptospirosis. Wild mammals infected with Leptospirosis were caught in Japan [66]. 

This study demonstrates the severe consequence of LULCC over Wayanad (case study area) by replacing forest with plantations (forest and agriculture plantation). As the world has just experienced a pandemic, and as the expansion of plantation continues unabated the pathways of outbreak of EIDs due to LULCC should receive attention. For example, the evidences of Dengue and Leptospirosis outbreak are apparent in Wayanad due to the deforestation through this study, the pathway of the outbreak of these diseases needs further investigation. We acknowledge that the impact of LULCC on the disease transmission via the changes in vector population dynamics should be studied through modelling framework. We also acknowledge that climate change may have an impact on the outbreak of EIDs, and this needs a thorough investigation as well. Plantation not only causes the loss of biodiversity, but also poses a fatal threat in the community.

## Figures and Tables

**Table 1:** Land use and land cover (LULC) of Wayanad district from 1950–2018 (area in sqkm).

Land Cover Type	1950	1982	2012	2018
Forest	1811.35	1064.68	888.84	672.89
Cultivation	255.72	530.48	225.29	179.57
Plantation (agriculture and forest)	63.93	529.99	913.204	1215.15
Others	N.A *	N.A *	107.97 *	87.23

* For 1950 and 1982, no data was available for built up, grazing, barren, water bodies for 2012, these data were available and categorized under ‘Others’.

**Table 2:** Percentage of land use and land cover (LULC) in each forest division in 2018.

	North Wayanad	South Wayanad	Wayanad Wildlife Division
Forest in %	30.81	20.01	64.27
Plantation (forest & agricultural) in %	57.45	63.84	33.04
High altitude grassland in %	2.71	4.29	0
Crop land (Paddy field & fallow) in %	8.63	10.13	2.62
Others (built up, waterbody) in %	0.39	1.71	0.06
Total	100	100	100

**Table 3:** List of confirmed cases of three selected emerging infectious diseases (EIDs) in Wayanad district.

	2011	2012	2013	2014	2015	2016	2017	2018	2019	2020
Dengue Fever	8	4	50	44	157	217	463	48	117	49
Leptospirosis	48	65	23	78	137	112	101	114	83	158
KFD			1	1	102	9	0	0	7	29

## Supplementary Materials

The following supporting information can be downloaded at: https://www.mdpi.com/article/10.3390/ijerph19127036/s1.
