# Supplementary material for: Impact of Plantation Induced Forest Degradation on the Outbreak of Emerging Infectious Diseases—Wayanad District, Kerala, India
Source: Int J Environ Res Public Health. 2022 Jun 8;19(12):7036. doi: 10.3390/ijerph19127036 (PMC9222524; doi:10.3390/ijerph19127036)
Supplement: Supplementary file 1 [file ijerph-19-07036-s001.zip › SuppIementary table&questionnair.pdf]

Supporting Information for

Impact of Plantation Induced Forest Degradation on the Outbreak of Emerging Infectious Diseases - Wayanad district, Kerala, India.

Kakoli Saha[1], Debjani Ghatak[2], and Nair Shruti S. Muralee[3]

[1]School of Planning and Architecture, Bhopal, India

[2]Department of Geography, Texas A&M University, College Station, TX, USA

[3] Madhya Pradesh Rurban Mission, Government of India

Corresponding author: Kakoli Saha (kakolisaha@spabhopal.ac.in)

Contents of this file

Tables S1

Introduction

The supporting table and figures provided here to show that results mentioned in the main text for a peak year is also true for other years.

Table S1. Number of cases registered for human monkey conflict

Year	No. of Cases
2013	300
2014	390
2015	484
2016	430
2017	480
2018	440
2019	300

Questionnaire to authorities and experts:

Name: Age:
Gender:
Designation: Qualification: Experience

Q1. How landuse change take place? What NOC or procedure has to be examined/considered to change the forest landuse?

Q2. What do you think is the main reason for infectious disease outbreak or communicable diseases especially through animals and insects?

Q3. What do you think is the main reason of deforestation in Wayanad district, Kerala?

Q4. Which sector plays major role in cutting down forest? Is it real estate, plantations, industries, mining, dam construction or other factors?

Q5. Do you think deforestation can be a reason for increasing communicable diseases in Kerala?

Q6. Do you think loss of habitat of animals bring them in contact with humans leading to more cases/ danger to human health?

Q7. Who are the most vulnerable to health risk and diseases outbreaks?
