# Supplementary figures and images for: Impact of Plantation Induced Forest Degradation on the Outbreak of Emerging Infectious Diseases—Wayanad District, Kerala, India
Source: Int J Environ Res Public Health. 2022 Jun 8;19(12):7036. doi: 10.3390/ijerph19127036 (PMC9222524; doi:10.3390/ijerph19127036)

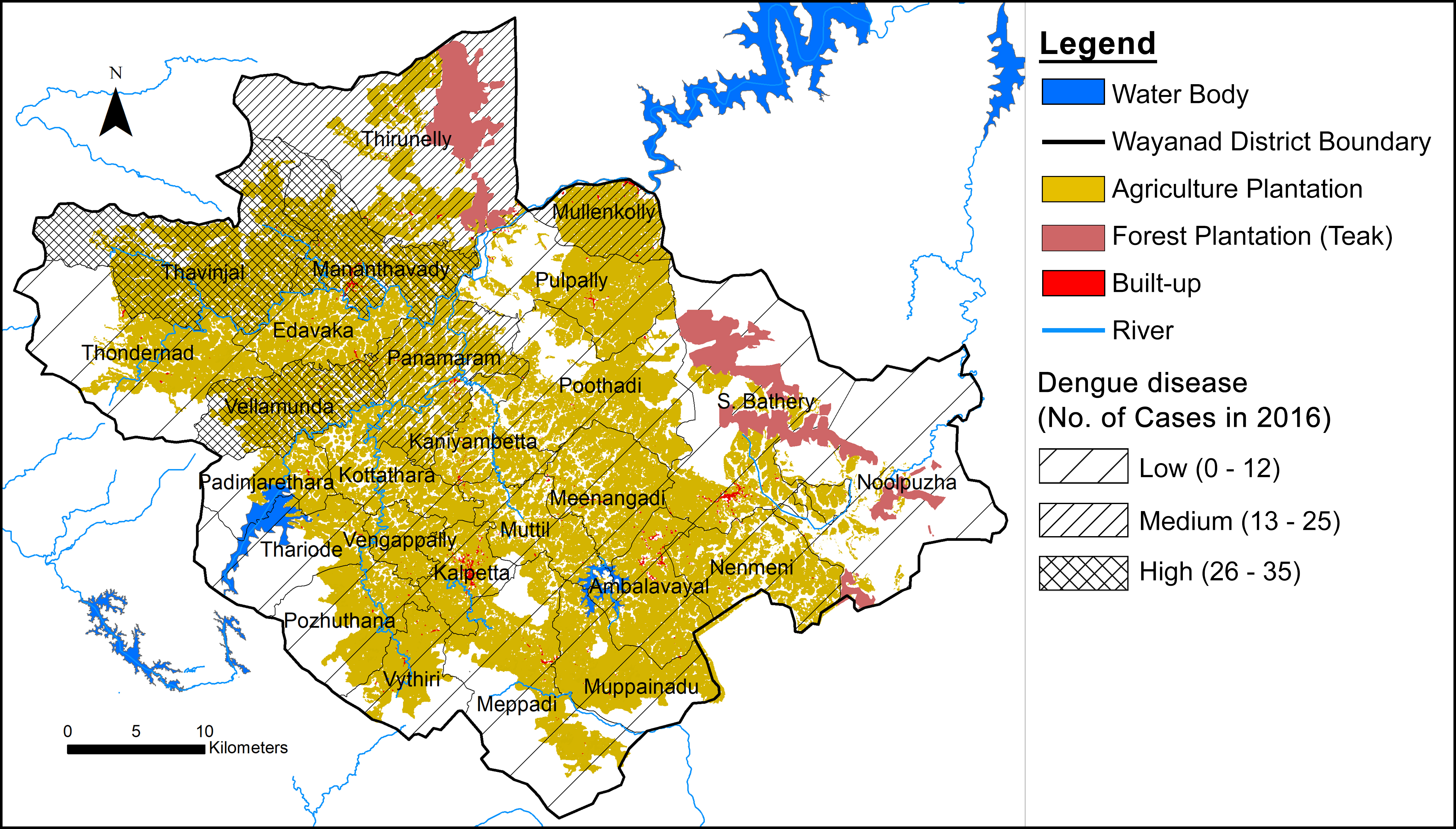

Supplement: Supplementary file 1 [file ijerph-19-07036-s001.zip › dengue_2016_N1.tif.jpg]

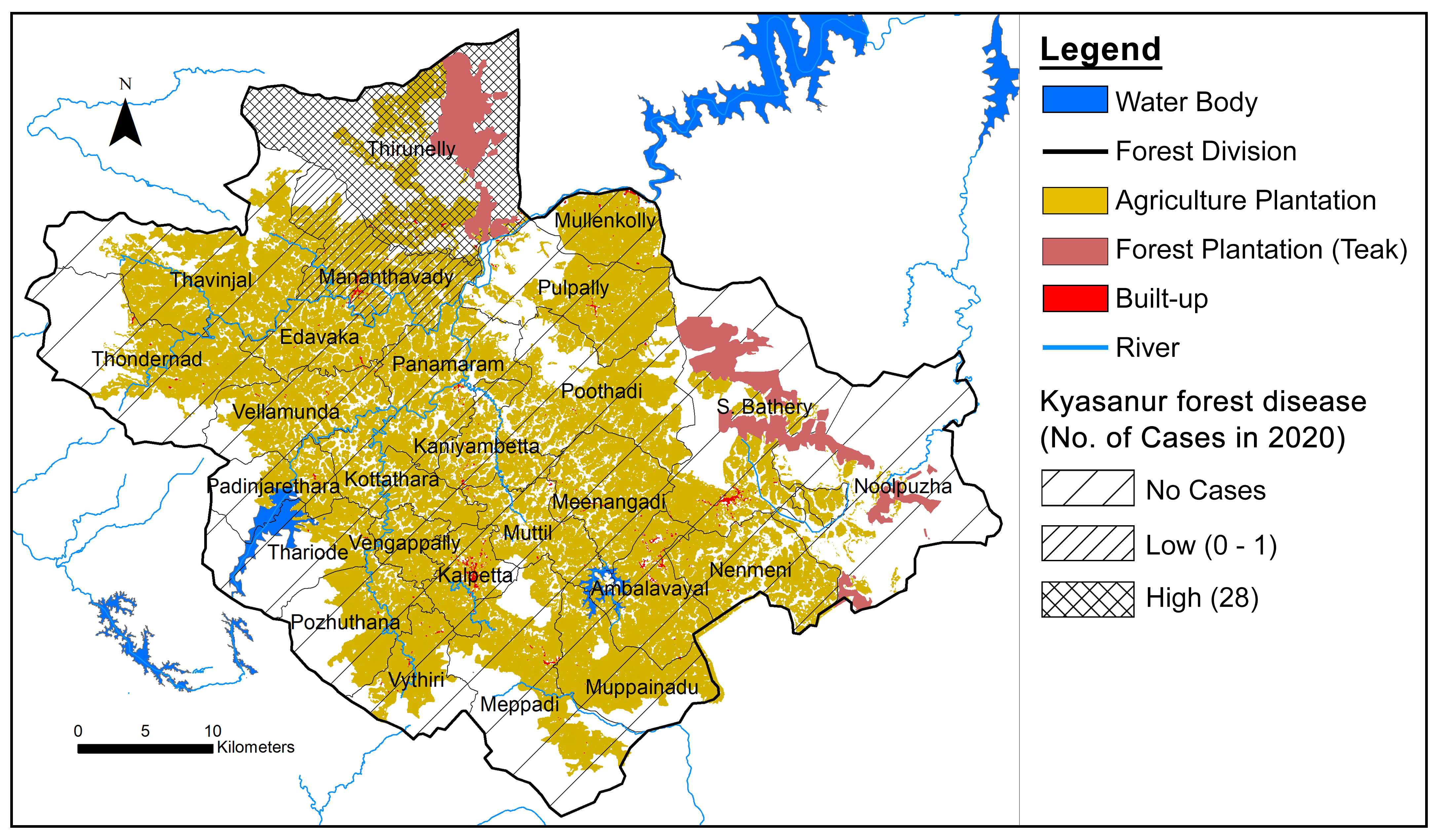

Supplement: Supplementary file 1 [file ijerph-19-07036-s001.zip › kfd 2020.jpg]

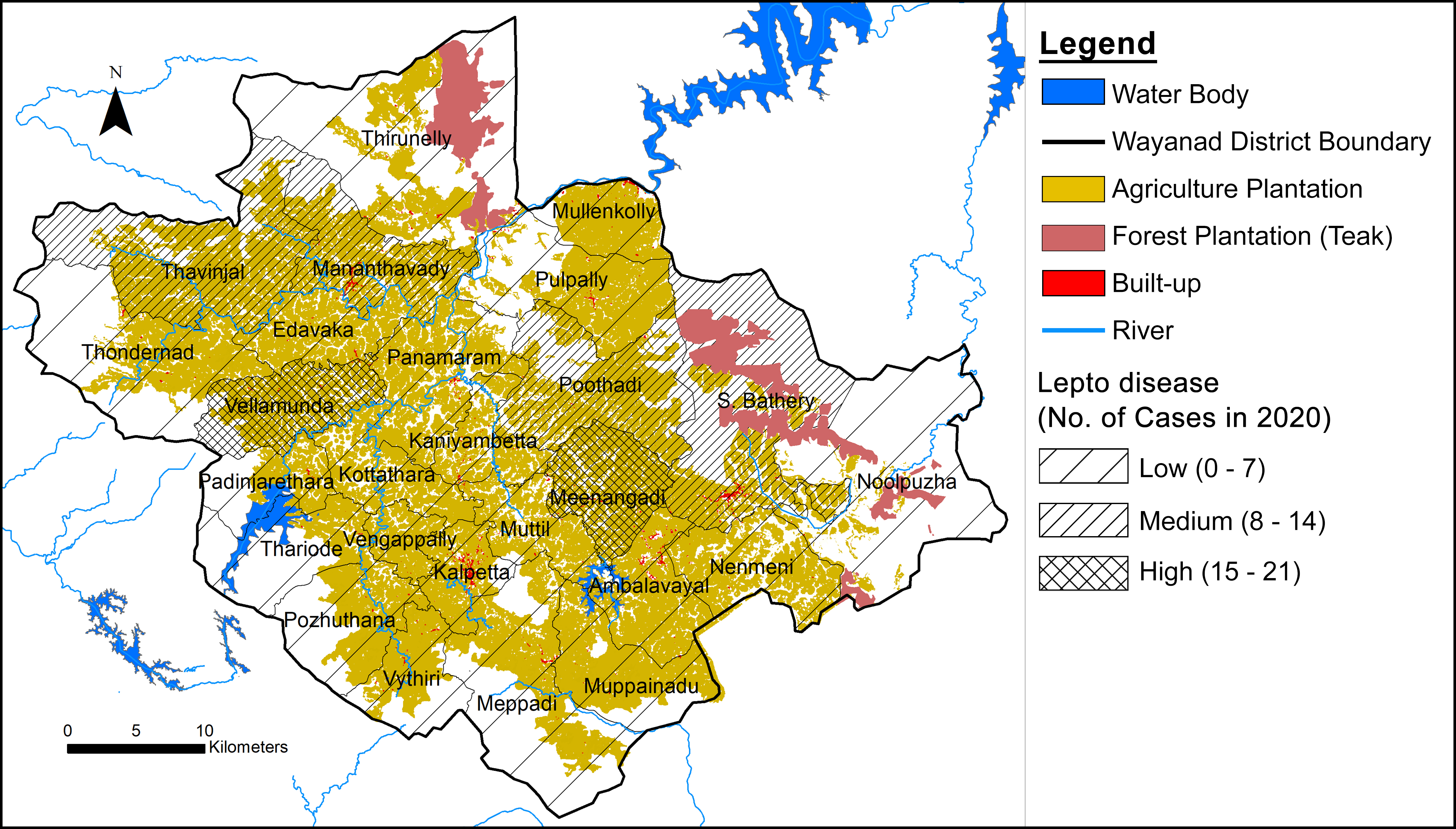

Supplement: Supplementary file 1 [file ijerph-19-07036-s001.zip › lepto_2020_N1.tif.jpg]
